# Impact of SGLT2 Inhibitors on Cardiovascular Risk Scores, Metabolic Parameters, and Laboratory Profiles in Type 2 Diabetes

**DOI:** 10.3390/life15050722

**Published:** 2025-04-29

**Authors:** Nazif Yalçın, Selman Aktaş, Seyit Uyar, Nizameddin Koca

**Affiliations:** 1 Department of Internal Medicine, Bursa Faculty of Medicine, University of Health Sciences, Bursa City Training and Research Hospital, Bursa 16009, Türkiye; nazifyalcin16@gmail.com; 2 Department of Biostatistics, Hamidiye Faculty of Medicine, University of Health Sciences, Istanbul 34396, Türkiye; selmanakts@gmail.com; 3 Department of Internal Medicine, University of Health Sciences, Antalya Training & Research Hospital, Antalya 07058, Türkiye; seyituyar79@hotmail.com

**Keywords:** SGLT2 inhibitors, Type 2 diabetes mellitus, cardiovascular risk, SCORE2-DM, metabolic regulation, cardiometabolic management, glycemic control

## Abstract

**Background**: Cardiovascular disease (CVD) is a leading cause of mortality in Type 2 diabetes mellitus (T2DM). Sodium-glucose co-transporter 2 (SGLT2) inhibitors are known to provide cardioprotective effects, but their influence on validated cardiovascular risk models remains underexplored. This study assessed the impact of SGLT2 inhibitors on cardiovascular risk scores, metabolic parameters, and laboratory profiles over six months. **Methods**: This study was conducted on 152 T2DM patients initiating SGLT2 inhibitors. Cardiovascular risk was evaluated using the SCORE2-DM model at baseline and after six months. Generalized Estimating Equation (GEE) analysis assessed temporal risk stratification changes. Metabolic parameters and laboratory profiles were analyzed using repeated-measures ANOVA. **Results**: Cardiovascular risk scores decreased significantly, i.e., from 21.68 to 17.43 (*p* < 0.001). Systolic and diastolic blood pressure were reduced by 9.21 mmHg and 6.16 mmHg, respectively (*p* < 0.001). BMI declined by 1.27 kg/m^2^ (*p* < 0.001), and HbA1c decreased by 1.38% (*p* < 0.001). Triglyceride levels dropped by 22.91 mg/dL (*p* < 0.001), while renal parameters remained stable. The GEE analysis confirmed significant shifts to lower cardiovascular risk categories (β = −0.777, *p* < 0.001), with comparable efficacy between empagliflozin and dapagliflozin (*p* = 0.922). **Conclusions**: SGLT2 inhibitor therapy significantly reduces cardiovascular risk and improves metabolic and laboratory parameters in T2DM patients. These findings highlight the importance of integrating SGLT2 inhibitors into comprehensive cardiometabolic management strategies.

## 1. Introduction

Type 2 diabetes mellitus (T2DM) is an increasingly prevalent public health issue, responsible for approximately 1.5 million deaths annually [1]. In the management of T2DM, achieving optimal blood glucose regulation can reduce the risk of numerous micro- and macrovascular complications, including cardiovascular disease (CVD), diabetic nephropathy, diabetic retinopathy, and mortality [2]. However, medications used in T2DM treatment may have side effects such as weight gain and hypoglycemia, which can undermine the desired reduction in cardiovascular complication risks [3].

These drugs, also known as gliflozins or glucoretics, reduce glucose reabsorption in the renal proximal tubules, thereby lowering blood glucose levels [4]. In diabetic patients, the glucose excretion threshold is elevated due to glucose-mediated proximal tubule hypertrophy and increased SGLT2 expression [5]. These changes result in enhanced sodium (Na^+^) reabsorption, increased caloric retention, volume expansion, and subsequent hypertension [6,7]. By inhibiting SGLT2, the tubular maximum reabsorption rate is reduced, increasing urinary glucose excretion and lowering plasma glucose concentrations [8].

Inflammation and oxidative stress play significant roles in the development of complications in T2DM patients [9,10]. In particular, inflammatory processes contribute to both the onset and progression of coronary artery disease. SGLT2 inhibitors have been shown to improve inflammatory profiles in diabetic patients [11,12]. These findings highlight the anti-inflammatory properties of SGLT2 inhibitors [12].

Beyond glucose and weight reduction, SGLT2 inhibitors have been shown to lower blood pressure (systolic by 3–5 mmHg and diastolic by 2–3 mmHg) without causing hypotension [13]. Studies have demonstrated that SGLT2 inhibitors improve blood pressure regulation and are associated with fewer hospitalizations and better outcomes in patients with a history of cardiovascular disease [14,15]. Pathophysiologically, there is a strong interplay between kidney dysfunction and heart failure [16]. As kidney disease progresses, volume overload due to impaired fluid excretion exacerbates heart failure. By reducing glomerular stress and volume overload, SGLT2 inhibitors preserve or improve kidney function, which benefits cardiac health [17]. These agents promote mild osmotic diuresis and natriuresis, improving filling conditions and reducing systemic sodium content, thereby protecting against heart failure [18,19]. Additionally, SGLT2 inhibitors enhance cardiac energy efficiency by increasing circulating ketone bodies, which may help preserve cardiac function in patients with reduced ejection fraction [20]. The oxidation of ketones is widely believed to improve outcomes in heart failure patients [21]. The sympathetic nervous system (SNS) plays a critical role in the pathogenesis of heart failure, and therapies that reduce SNS activity have been shown to improve outcomes. There is both direct and indirect evidence that SGLT2 inhibitors reduce SNS activity. A recent study investigated the interaction between renal SGLT2 inhibition and SNS activity, demonstrating that chemical denervation in BPH/2J mice reduced SGLT2 protein expression in the kidney [22,23]. Thus, SGLT2 inhibitors may attenuate SNS activity by reducing afferent sympathetic nerve stimulation.

SGLT2 inhibitors can reduce cardiovascular risk through various mechanisms. The American Heart Association (AHA) and the European Society of Cardiology (ESC) recommend using SCORE risk scoring systems to evaluate 10-year absolute cardiovascular risk [24,25]. In this study, our objective is to investigate the influence of SGLT2 inhibitors on cardiovascular risk by performing SCORE2 calculations in patients receiving these agents. To the best of our knowledge, this is the first study specifically assessing the effect of SGLT2 inhibitors on cardiovascular risk stratification using the SCORE2-DM model in patients with Type 2 diabetes mellitus.

## 2. Methods

### 2.1. Ethical Considerations

The study was approved by the Institutional Review Board (21.08.2024 decision no: 2024-13/2) and conducted in accordance with the Declaration of Helsinki. Written informed consent was obtained from all participants before enrollment.

### 2.2. Study Design and Setting

This observational study was conducted to evaluate the effects of SGLT2 inhibitor therapy on cardiovascular risk, metabolic parameters, and other laboratory values over 6 months. The study was conducted at a tertiary care center specializing in diabetes management and cardiovascular risk reduction.

### 2.3. Study Population

A total of 152 patients with Type 2 diabetes mellitus (T2DM) who were initiated on SGLT2 inhibitors as part of their standard care were included in the study. Empagliflozin and dapagliflozin were selected based on their availability and clinical use in our national healthcare system. Other SGLT2 inhibitors, such as canagliflozin, were not included due to the lack of local regulatory approval and accessibility. Inclusion criteria were as follows: age between 18 and 80 years, a confirmed diagnosis of T2DM based on ADA criteria, and baseline HbA1c levels ≥ 7.0%. Exclusion criteria included severe renal impairment (eGFR < 30 mL/min/1.73 m^2^), active malignancy, pregnancy or lactation, use of other antidiabetic agents introduced during the study period, and any changes in dosage of cardiovascular medications, including antihypertensives, diuretics, and statins.

### 2.4. Data Collection and Measurements

Baseline demographic data, clinical parameters, and laboratory values were collected during SGLT2 inhibitor initiation (0 month). Follow-up data were obtained in the third month and sixth month after initiation. Key variables included weight, body mass index (BMI), systolic blood pressure (SBP), diastolic blood pressure (DBP), fasting blood glucose (FBG), HbA1c, lipid profile (triglycerides, LDL, HDL), renal function markers (creatinine, urea), and liver enzymes (AST, ALT). Cardiovascular risk scores were calculated using the SCORE-2 DM model. All measurements were performed under standardized clinical and laboratory protocols.

### 2.5. SCORE-2 DM Calculation

The SCORE-2 DM risk score was calculated using patient-specific data, including age, sex, smoking status, systolic blood pressure, total cholesterol, and high-density lipoprotein (HDL) cholesterol levels. The model incorporated additional adjustments for the presence of diabetes, providing a refined estimate of 10-year cardiovascular risk for individuals with diabetes. Risk stratification was performed based on SCORE-2 DM thresholds: low risk (<10%), moderate risk (10–19%), high risk (20–29%), and very high risk (≥30%) [26]. This stratification allowed for detailed subgroup analysis to evaluate the impact of SGLT2 inhibitor therapy on patients at varying levels of baseline cardiovascular risk. Each patient’s risk score was calculated at baseline (0 month) and reassessed at six months to evaluate changes attributable to SGLT2 inhibitor therapy.

### 2.6. Intervention

Patients received continuous SGLT2 inhibitor therapy (empagliflozin or dapagliflozin) as part of their antidiabetic regimen for 6 months, with clinical and laboratory assessments conducted at baseline, 3 months, and 6 months. To ensure isolated evaluation of the SGLT2 inhibitor effects, no adjustments were made to patients’ existing antihyperlipidemic, antidiabetic (excluding the newly initiated SGLT2i), or antihypertensive treatments during the study period. While drug regimens remain constant, we acknowledge that cardiovascular drugs such as antihypertensives may also have metabolic effects [27]. Patients requiring additional medical interventions during follow-up were excluded from the study.

### 2.7. Outcomes

The primary outcome was the change in cardiovascular risk scores (SCORE-2 DM) over six months. Secondary outcomes included changes in anthropometric measures (weight, BMI), blood pressure, glycemic control (FBG, HbA1c), lipid profile, and renal and hepatic parameters.

### 2.8. Statistical Analysis

Data normality was assessed using the Shapiro-Wilk test. Continuous variables with normal distributions were expressed as mean ± standard deviation (SD), while non-normally distributed variables were reported as median (Min–Max). Categorical variables were summarized as frequencies and percentages.

For longitudinal comparisons of continuous outcomes across three-time points (baseline, third month, and sixth month), repeated-measures analysis of variance (ANOVA) with Greenhouse-Geisser correction was applied to address violations of sphericity in normally distributed data. Nonparametric longitudinal analyses were conducted using the Friedman test for non-normally distributed variables. Post hoc pairwise comparisons with Bonferroni adjustment were performed to control for Type I error inflation in parametric and nonparametric frameworks.

Categorical variable dynamics, including shifts in cardiovascular risk stratification (low, moderate, high, very high), were analyzed using the McNemar-Bowker test for paired nominal proportions.

### 2.9. Generalized Estimating Equation (GEE) Modeling

An ordinal logistic GEE model was constructed to account for intra-subject correlations in repeated measurements and assess temporal changes in SCORE2-DM risk categories. The model incorporated an exchangeable working correlation matrix to accommodate within-patient dependency across time points. Covariates included treatment phase (baseline vs. follow-up), drug type (empagliflozin vs. dapagliflozin), and their interaction terms.

Parameter estimates were reported as β coefficients with robust standard errors (SE) and 95% confidence intervals (CI). Wald χ^2^ statistics were computed to test the significance of fixed effects, with interaction terms being retained in the model regardless of significance to ensure hierarchical testing validity. Missing data were addressed under the missing-at-random (MAR) assumption using full-information maximum likelihood estimation.

All analyses were performed using SPSS version 26.0 (IBM Corp., Armonk, NY, USA) with α = 0.05 defining statistical significance for two-tailed hypotheses. Effect sizes are reported where applicable to complement null hypothesis testing.

### 2.10. Sample Size Determination

The sample size was calculated based on a minimum detectable change in cardiovascular risk scores with an alpha of 0.05 and a power of 80%. Accounting for a potential dropout rate of 10%, 150 patients were required to achieve statistical significance.

## 3. Results

The demographic and laboratory values of patients at baseline (0 months), three months, and six months following SGLT2 inhibitor treatment are presented in Table 1. The mean age of the study population was 58.47 ± 9.86 years, with 52.6% being male. Most of the cohort had primary education (59.9%), and 63.2% were active smokers.

### 3.1. Anthropometric and Blood Pressure Changes

Significant reductions were observed in weight, body mass index (BMI), systolic blood pressure (SBP), and diastolic blood pressure (DBP) across the follow-up period. Mean weight decreased from 86.57 ± 11.43 kg at baseline to 84.88 ± 10.75 kg at three months and further to 83.01 ± 9.59 kg at six months (*p* < 0.001 for all comparisons). BMI was consistently reduced, from 31.59 ± 4.45 kg/m^2^ at baseline to 30.99 ± 4.33 kg/m^2^ at three months and 30.32 ± 4.04 kg/m^2^ at six months (*p* < 0.001 for all comparisons).

SBP decreased significantly from 131.64 ± 8.15 mmHg at baseline to 126.43 ± 6.93 mmHg at three months and 122.43 ± 8.14 mmHg at six months (*p* < 0.001 for all comparisons). Similarly, DBP reduced from 82.04 ± 6.49 mmHg to 77.53 ± 6.18 mmHg at three months and 75.88 ± 6.82 mmHg at six months (*p* < 0.001 for baseline vs. three months and six months; *p* = 0.003 for three months vs. six months).

### 3.2. Glycemic Control and Lipid Profile

There was a marked improvement in glycemic control over the study period. Fasting blood glucose (FBG) decreased from 218.78 ± 76.4 mg/dL at baseline to 180.22 ± 49.03 mg/dL at three months and 162.39 ± 35.27 mg/dL at six months (*p* < 0.001 for all comparisons). Hemoglobin A1c (HbA1c) also declined significantly from 9.1 ± 1.6% at baseline to 8.26 ± 1.25% at three months and 7.72 ± 1.04% at six months (*p* < 0.001 for all comparisons).

Triglyceride levels reduced significantly, from 173.84 ± 57.6 mg/dL at baseline to 159.23 ± 47.81 mg/dL at three months and 150.93 ± 37.13 mg/dL at six months (*p* < 0.001 for all comparisons). Low-density lipoprotein (LDL) cholesterol also decreased from 126.07 ± 29.07 mg/dL at baseline to 116.23 ± 23.04 mg/dL at three months and 111.8 ± 21.43 mg/dL at six months (*p* < 0.001 for all comparisons). High-density lipoprotein (HDL) levels showed a slight increase from 43.93 ± 8.58 mg/dL at baseline to 44.42 ± 8.02 mg/dL at three months and 44.46 ± 7.49 mg/dL at six months, though these changes were not statistically significant (*p* > 0.05).

### 3.3. Liver and Renal Function

A modest but statistically significant reduction was observed in aspartate aminotransferase (AST) levels, from 35.01 ± 9.87 IU/L at baseline to 33.52 ± 7.88 IU/L at three months and 32.21 ± 6.63 IU/L at six months (*p* < 0.001 for baseline vs. three months and six months). Alanine aminotransferase (ALT) levels showed a slight increase at three months (34.34 ± 6.1 IU/L) but decreased by six months (34.62 ± 5.8 IU/L), with significant changes between baseline and six months (*p* = 0.014 for baseline vs. three months; *p* = 0.001 for baseline vs. six months).

Creatinine levels increased slightly from 0.89 ± 0.14 mg/dL at baseline to 0.96 ± 0.17 mg/dL at three months and 0.95 ± 0.22 mg/dL at six months (*p* < 0.001 for baseline vs. three months and six months). Urea levels did not change significantly across the study period (*p* > 0.05).

### 3.4. Transition Analysis of Demographic and Laboratory Values (Table 2)

Over the six months, weight decreased by 1.69 kg (−1.95%) between the third and zeroth months, by 1.87 kg (−2.2%) between the sixth and third months, and by a total of 3.56 kg (−4.11%) over the entire study period. BMI followed a similar trend, with reductions of 0.6 kg/m^2^ (−1.9%), 0.67 kg/m^2^ (−2.16%), and 1.27 kg/m^2^ (−4.02%) for the same intervals, respectively. SBP decreased by 5.21 mmHg (−3.96%) between the third and zeroth months, by 4 mmHg (−3.16%) between the sixth and third months, and by 9.21 mmHg (−7%) overall. DBP also exhibited significant reductions, decreasing by 4.51 mmHg (−5.5%), 1.65 mmHg (−2.13%), and 6.16 mmHg (−7.51%) for the respective intervals.

**Table 2:** Demographic and laboratory values transition within the six months.

	3rd–0th	6th–3rd	6th–0th
**Weight, kg, Δ (%)**	−1.69 (−1.95)	−1.87 (−2.2)	−3.56 (−4.11)
**BMI, kg/m^2^, Δ (%)**	−0.6 (−1.9)	−0.67 (−2.16)	−1.27 (−4.02)
**SBP, mmHg, Δ (%)**	−5.21 (−3.96)	−4.0 (−3.16)	−9.21 (−7)
**DBP, mmHg, Δ (%)**	−4.51 (−5.5)	−1.65 (−2.13)	−6.16 (−7.51)
**HbA1c, %, Δ (%)**	−0.84 (−9.23)	−0.54 (−6.54)	−1.38 (−15.16)
**FBG, mg/dL, Δ (%)**	−38.56 (−17.63)	−17.83 (−9.89)	−56.39 (−25.77)
**Urea, mg/dL, Δ (%)**	0.34 (1.01)	0.02 (0.06)	0.36 (1.06)
**Creatinine, mg/dL, Δ (%)**	0.07 (7.87)	−0.01 (−1.04)	0.06 (6.74)
**AST, IU/L, Δ (%)**	−1.49 (−4.26)	−1.31 (−3.91)	−2.8 (−8)
**ALT, IU/L, Δ (%)**	0.79 (2.35)	0.28 (0.82)	1.07 (3.19)
**Triglycerides, mg/dL, Δ (%)**	−14.61 (−8.4)	−8.3 (−5.21)	−22.91 (−13.18)
**HDL, mg/dL, Δ (%)**	0.49 (1.12)	0.04 (0.09)	0.53 (1.21)
**LDL, mg/dL, Δ (%)**	−9.84 (−7.81)	−4.43 (−3.81)	−14.27 (−11.32)

BMI: body mass index, SBP: systolic blood pressure, DBP: diastolic blood pressure, FBG: fasting blood glucose, AST: aspartate transaminase, ALT: alanine transaminase, HDL: high-density lipoprotein, LDL: low-density lipoprotein.

Glycemic parameters showed marked improvement, with HbA1c decreasing by 0.84% (−9.23%) between the third and zeroth months, by 0.54% (−6.54%) between the sixth and third months, and by 1.38% (−15.16%) over the entire study period. Fasting blood glucose (FBG) decreased significantly by 38.56 mg/dL (−17.63%) between the third and zeroth months, by 17.83 mg/dL (−9.89%) between the sixth and third months, and by 56.39 mg/dL (−25.77%) overall.

Renal and hepatic parameters showed more modest changes. Urea levels increased slightly by 0.34 mg/dL (1.01%) between the third and zeroth months, by 0.02 mg/dL (0.06%) between the sixth and third months, and by 0.36 mg/dL (1.06%) overall. Creatinine levels increased by 0.07 mg/dL (7.87%) between the third and zeroth months, decreased slightly by 0.01 mg/dL (−1.04%) between the sixth and third months, and showed an overall increase of 0.06 mg/dL (6.74%). AST decreased by 1.49 IU/L (−4.26%), 1.31 IU/L (−3.91%), and 2.8 IU/L (−8%) for the respective intervals, while ALT increased slightly by 0.79 IU/L (2.35%), 0.28 IU/L (0.82%), and 1.07 IU/L (3.19%) overall.

Lipid parameters also improved, with triglycerides decreasing by 14.61 mg/dL (−8.4%) between the third and zeroth months, by 8.3 mg/dL (−5.21%) between the sixth and third months, and by 22.91 mg/dL (−13.18%) overall. HDL levels showed modest increases of 0.49 mg/dL (1.12%), 0.04 mg/dL (0.09%), and 0.53 mg/dL (1.21%) for the respective intervals. LDL levels decreased by 9.84 mg/dL (−7.81%) between the third and zeroth months, by 4.43 mg/dL (−3.81%) between the sixth and third months, and by 14.27 mg/dL (−11.32%) overall.

### 3.5. Cardiovascular Risk Score

The SCORE-2 DM cardiovascular risk score significantly reduced from 21.68 ± 9.83 at baseline to 17.43 ± 8.72 at sixth months (*p* < 0.001). This indicates a notable decrease in cardiovascular risk associated with SGLT2 inhibitor use over the study period.

### 3.6. Cardiovascular Risk Transition

Analysis of cardiovascular risk category transitions revealed significant changes between the baseline and the final measurement. At baseline, five patients (initially categorized as low-risk) remained in the low-risk group at the final measurement. Among patients categorized as moderate at baseline, 3 transitioned to low risk, and 11 remained at moderate risk. High-risk patients exhibited notable transitions, with 17 moving to moderate risk and 36 remaining high risk. Of the very high-risk patients at baseline, 28 transitioned to high risk, while 52 remained very high-risk. The overall shifts in risk categories were statistically significant (χ^2^ = 48.00, *p* < 0.001, Table 3).

Post hoc analyses comparing the first and last measurements revealed significant differences between specific cardiovascular risk categories. Specifically, transitions from moderate to high risk and from high to very high risk were statistically significant (*p* < 0.001 for both comparisons). No significant differences were observed in other pairwise comparisons, including low risk to moderate risk, low risk to high risk, and low risk to very high risk (*p* > 0.05 for all). Detailed adjusted *p*-values for these comparisons are presented in Table 4.

### 3.7. Very-High Risk and High-Moderate Risk Comparisons

A detailed analysis of the very-high and high-moderate risk groups at baseline and the sixth month revealed statistically significant transitions. At baseline, 36 patients were categorized as high risk, all of whom remained at high risk by the sixth month. In contrast, 28 patients initially categorized as very high risk transitioned to high risk, while 52 remained in the very-high risk category (χ^2^ < 0.001). For moderate-risk patients, 11 transitioned to high risk by the sixth month, and 17 high-risk patients moved to moderate risk (χ^2^ < 0.001). These results are detailed in Table 5.

**Figure 1:**
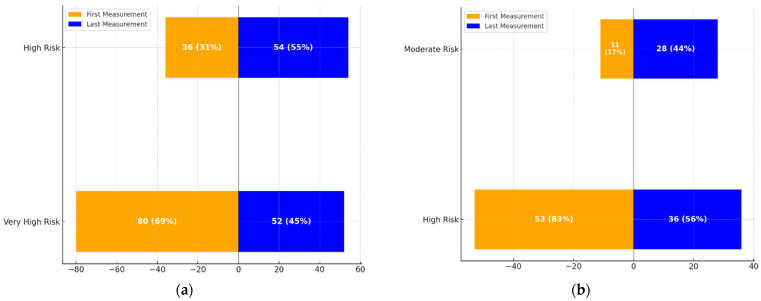
(**a**): Transition dynamics between High-Risk and Very High-Risk categories over the treatment period. The figure shows the proportion of patients in each category at the first and last measurements. (**b**): Transition dynamics between Moderate-Risk and High-Risk categories over the treatment period. The figure highlights the changes in the number and percentage of patients transitioning between these categories at the first and last measurements.

The transitions in cardiovascular risk categories over time are illustrated in Figure 1.

- **Figure 1a**: This graph demonstrates the transition dynamics between the High-Risk and Very High-Risk categories. The proportion of individuals in the High-Risk group increased from 36 (31%) to 54 (55%), while those in the Very High-Risk group decreased from 80 (69%) to 52 (45%).
- **Figure 1b**: This graph highlights transitions between Moderate and High-Risk categories. The Moderate-Risk group expanded from 11 (17%) to 28 (44%), while the High-Risk group saw a reduction from 53 (83%) to 36 (56%).

These visualizations underscore the significant changes in cardiovascular risk profiles over the treatment period, aligning with the statistical findings.

### 3.8. General Estimating Equation (GEE)

The GEE analysis evaluating changes in SCORE2-DM risk categories before and after SGLT2 inhibitor treatment is presented in Table 6. The model demonstrated significant overall effects for measurement time (before vs. after treatment; Wald χ^2^ = 10.624, *p* < 0.001), but no significant effects for drug type (empagliflozin vs. dapagliflozin; Wald χ^2^ = 0.010, *p* = 0.922) or measurement-drug interactions (Wald χ^2^ = 1.462, *p* = 0.227).

Threshold parameters revealed distinct shifts in cardiovascular risk stratification. A significant reduction in high-risk categorization (β = −0.777, SE = 0.219, 95% CI (−1.206, −0.347), *p* < 0.001) and low-risk categorization (β = −0.597, SE = 0.220, 95% CI (−1.029, −0.165), *p* = 0.007) relative to the reference very-high-risk category was observed post-treatment. In contrast, moderate-risk categorization showed no statistically significant change (β = −0.026, SE = 0.232, 95% CI (−0.482, 0.429), *p* = 0.910).

Score 2-DM reference category: (Very High Risk).

The main effect of measurement time demonstrated a robust treatment-associated risk reduction (β = −0.724, SE = 0.222, 95% CI (−1.159, −0.288), *p* = 0.001). However, neither empagliflozin (β = −0.031, SE = 0.317, 95% CI (−0.652, 0.590), *p* = 0.922) nor dapagliflozin showed differential effects on risk stratification when compared directly. Interaction terms between measurement time and drug type were non-significant (*p* > 0.05 for all comparisons), indicating comparable efficacy between the two SGLT2 inhibitors in modulating SCORE2-DM outcomes.

These results collectively suggest that SGLT2 inhibitor therapy significantly reduces cardiovascular risk profiles as quantified by SCORE2-DM, independent of specific drug selection.

## 4. Discussion

The initiation of SGLT2 inhibitor therapy in patients with T2DM resulted in significant improvements across various clinical parameters. Over the six-month study period, there was a marked reduction in cardiovascular risk scores (SCORE2-DM), from 21.68 to 17.43, accompanied by notable decreases in body mass index (BMI), systolic and diastolic blood pressure, FBG, and HbA1c. Triglyceride levels also decreased significantly, while renal parameters remained stable despite osmotic diuresis. The Generalized Estimating Equation (GEE) analysis confirmed these improvements, showing a statistically significant shift in risk stratification toward lower-risk categories, with no difference in efficacy between empagliflozin and dapagliflozin. This study uniquely contributes to the current literature by being the first to evaluate and report significant reductions in SCORE2-DM cardiovascular risk scores associated with SGLT2 inhibitor therapy over a six-month period in patients with Type 2 diabetes mellitus.

SGLT2 inhibitors are widely used in treating T2DM. Their popularity stems from their ability to provide glycemic control while offering additional benefits such as a low risk of hypoglycemia, weight loss, improved blood pressure regulation, and renal and cardioprotective effects.

The activity of SGLT2 inhibitors is independent of insulin status, meaning their efficacy is not diminished by insulin resistance or absolute insulin deficiency, making them suitable for use in both Type 2 and Type 1 diabetes. Their glucose-lowering effect is more pronounced in individuals with higher blood glucose concentrations, and their hyperglycemia-dependent properties make them particularly useful in reducing postprandial glucose excursions. Meta-analyses of studies on SGLT2 inhibitors in T2DM have shown reductions in HbA1c of approximately 0.5% to 1% (6–11 mmol/mol) [28,29]. Due to their unique mechanism of action, SGLT2 inhibitors can be combined with other glucose-lowering agents, including insulin, and often reduce the required insulin dosage in both Type 2 and Type 1 diabetes [30,31]. In a study by Kamin M et al. [32] in Pakistan, the mean reduction in HbA1c was 1.1%. Similarly, Sohail E et al. [33] reported a mean HbA1c reduction of 1.34%. In a study by Prasanna Kumar KM [34] in India, patients using SGLT2 inhibitors experienced an average HbA1c reduction of 1.02%. In our study, patients treated with SGLT2 inhibitors showed a mean HbA1c reduction of 1.38%.

The mechanism of action of SGLT2 inhibitors involves increasing urinary glucose excretion, leading to caloric loss and subsequent weight reduction. Clinical trials in T2DM patients have generally reported weight loss of around 3 kg with SGLT2 inhibitors, though real-world observational studies lasting over a year have noted reductions exceeding 6 kg. This weight loss is primarily attributed to caloric loss through glucosuria, although reduced plasma volume may also contribute [35,36]. In the study by Sohail E et al. [33], patients using SGLT2 inhibitors experienced an average weight loss of 2.3 kg. Ghosh et al. [37] reported an average weight loss of 3.45 kg over 12 months in Indian patients using SGLT2 inhibitors. In our study, patients treated with SGLT2 inhibitors lost an average of 3.56 kg over six months, with 1.69 kg lost in the first three months and 1.87 kg in the subsequent three months.

SGLT2 inhibitors exert natriuretic effects, preventing excessive volume displacement. Consequently, they contribute to blood pressure reduction through increased sodium and water excretion. In a study by Mayne KJ et al. [38], systolic and diastolic blood pressure decreased by an average of 2.6 mmHg and 0.5 mmHg, respectively. Kamin M et al. [32] also reported significant blood pressure reductions, with systolic and diastolic blood pressure decreasing by 2.8 mmHg and 2.02 mmHg, respectively. Zanchi et al. [39] observed 5 mmHg and 2 mmHg reductions in systolic and diastolic blood pressure, respectively, in patients monitored over 24 h. Similarly, Georgianos et al. [40] reported reductions of 3.6 mmHg and 1.7 mmHg in systolic and diastolic blood pressure, respectively. In our study, blood pressure reductions were consistent with these findings, with systolic and diastolic blood pressure decreasing by 5.21 mmHg and 4.51 mmHg in the first three months and 4 mmHg and 1.65 mmHg in the subsequent three months. The more significant reduction observed in our study may be attributed to higher salt consumption in our population.

SGLT2 inhibitors are frequently preferred in patients with high renal and cardiac risk. Lipids play a significant role in cardiovascular disease risk and pathogenesis. The effects of SGLT2 inhibitors on lipid profiles remain controversial in the literature. A meta-analysis of 48 randomized trials found that SGLT2 inhibitors reduced triglyceride levels while increasing low-density lipoprotein (LDL) and high-density lipoprotein (HDL) levels [41]. Notably, many studies reported that increased LDL levels involved canagliflozin. A recent meta-analysis of 60 randomized trials involving 147,130 patients also found that SGLT2 inhibition increased LDL and HDL cholesterol while reducing triglyceride levels [42]. Additionally, newer SGLT2 inhibitors, such as remogliflozin, have been shown to increase HDL cholesterol levels significantly [43]. In a study by Premji R et al. [44], SGLT2 inhibitors were associated with modest increases in LDL and HDL cholesterol levels and significant reductions in triglycerides. The observed increase in LDL was attributed to larger, less atherogenic LDL particles. Differences in LDL changes may be due to reduced LDL receptor expression in liver parenchyma and decreased LDL uptake by hepatocytes [45]. In T2DM patients, 12 weeks of dapagliflozin treatment reduced the concentration of small, dense atherogenic LDL particles by 20% without affecting total LDL cholesterol levels [46]. Importantly, the SGLT2 inhibitors used in this study—empagliflozin and dapagliflozin—were selected based on their accessibility and regulatory approval status in our national healthcare system. Other agents, such as canagliflozin, were not included due to their unavailability in the local formulary. This choice reflects real-world prescribing practices and enhances the study’s relevance to routine clinical care within our setting, although it may limit direct comparisons with studies utilizing alternative SGLT2 inhibitors. In our study, consistent with the literature, patients exhibited significant reductions in triglyceride levels and modest increases in HDL levels. Notably, despite no changes in antihyperlipidemic therapy, LDL levels showed a modest decline, underscoring the need for further large-scale studies on the lipid-modifying effects of these drugs.

The glucosuric effects of SGLT2 inhibitors lead to caloric and weight loss, which may reduce hepatic steatosis [47]. A review of 11 studies involving 839 patients found that SGLT2 inhibitor users had lower alanine transaminase (ALT) and aspartate transaminase (AST) levels than controls [48]. Xu Z et al. [49] reported improved metabolic parameters and transaminase levels in patients using SGLT2 inhibitors. In our study, patients exhibited lower AST and ALT levels in the sixth month compared to baseline. The transient increase in ALT levels at the three-month measurement, which declined at six months, may reflect hepatic metabolic adjustments associated with glucosuria-induced caloric losses and related metabolic adaptations. Nevertheless, further long-term follow-up studies are required to elucidate these transient enzymatic elevations’ clinical significance and potential hepatic implications.

The GEE analysis revealed significant temporal reductions in cardiovascular risk categories over the six-month treatment period. Patients demonstrated a significant reduction in high-risk categorization (β = −0.777, *p* < 0.001) and low-risk categorization (β = −0.597, *p* = 0.007), with the overall effect of measurement time being highly significant (Wald χ^2^ = 10.624, *p* = 0.001). These findings suggest that SGLT2 inhibitor therapy effectively shifts patients into lower cardiovascular risk groups, which is consistent with reports that these agents reduce both systolic and diastolic blood pressure, improve glycemic control [13,50]. SGLT2 inhibitors are also associated with better outcomes in cardiovascular events [50,51].

Recent studies also support the sustained improvement in cardiovascular risk categories, indicating that SGLT2 inhibitors attenuate left ventricular remodeling and endothelial dysfunction, mechanisms that are critical for cardiovascular risk reduction [21,52]. Furthermore, reductions in inflammatory markers such as NLRP3 inflammasome activation have been linked to improved vascular health and reduced atherosclerotic progression, further substantiating the observed shifts in risk stratification [53].

**Figure 2:**
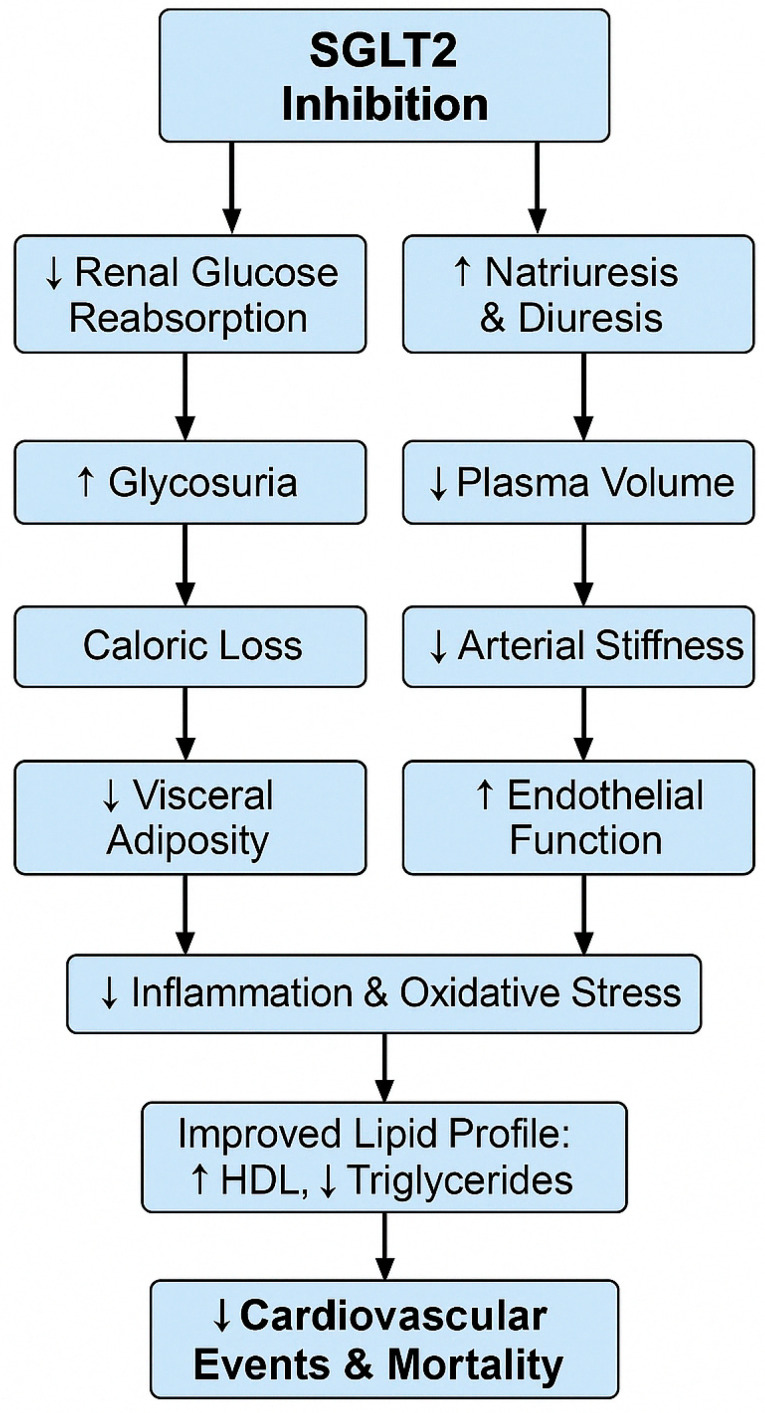
Proposed cardiometabolic mechanisms of SGLT2 inhibition. This schematic flowchart illustrates how SGLT2 inhibitors trigger renal glucose and sodium excretion, leading to downstream effects on visceral adiposity, insulin sensitivity, endothelial function, inflammation, lipid profile, and ultimately reduce cardiovascular events and mortality.

Several mechanisms may underlie the observed reduction in cardiovascular risk. SGLT2 inhibitors exert protective effects beyond glycemic control by modulating endothelial function, attenuating oxidative stress, and reducing low-grade inflammation. As shown in Figure 2, SGLT2 inhibition–induced glycosuria and natriuresis lead to caloric loss and plasma-volume changes, reducing visceral adiposity, enhancing insulin sensitivity and endothelial function, ultimately translating into lower cardiovascular events and mortality. This anti-inflammatory action is supported by studies demonstrating that SGLT2 inhibitors modulate the PI3K/Akt signaling pathway, a key regulator of insulin sensitivity and inflammatory homeostasis [54]. Furthermore, recent clinical and preclinical evidence indicates that SGLT2 inhibitors exert potent immunomodulatory effects by suppressing pro-inflammatory cytokines and chemokines, thereby significantly improving systemic metabolic outcomes [55]. These pathways may partially explain our cohort’s observed reductions in cardiovascular risk scores. These cardioprotective effects involve mitochondrial stabilization and suppression of pro-inflammatory pathways. SGLT2 inhibitors have been shown to enhance mitochondrial oxidative phosphorylation and ATP production while simultaneously reducing reactive oxygen species (ROS) generation, thereby playing a pivotal role in maintaining mitochondrial integrity and improving overall metabolic function [56,57].

Beyond currently available pharmacological options, future research may explore the synergistic potential of combining SGLT2 inhibitors with emerging therapeutic agents. For example, selenoproteins are established modulators of insulin sensitivity, and while their potential synergistic effects when combined with SGLT2 inhibitor therapy remain to be fully investigated, they represent a promising area for multi-dimensional metabolic research [58]. Additionally, extracellular vesicles derived from stem cells have shown promise in enhancing endothelial repair, modulating immune responses, and promoting tissue regeneration—pathways that align with the cardioprotective profile of SGLT2 inhibitors [59]. Integrating novel adjuvant therapies with SGLT2 inhibition could represent a forward-looking, multidimensional approach to metabolic and cardiovascular risk reduction.

Cardiovascular complications are among the most significant contributors to mortality in T2DM patients. Recent epidemiological data highlighted the strong association between T2DM and cardiovascular disease [60]. Since 2008, the FDA has required evidence of cardiovascular safety for new antihyperglycemic agents. Unexpectedly, studies such as EMPA-REG OUTCOME and DECLARE-TIMI demonstrated that SGLT2 inhibitors reduce mortality and morbidity [14,15]. Subsequent trials, including EMPEROR-Reduced and DAPA-HF, confirmed that empagliflozin and dapagliflozin reduce mortality and hospitalization in patients with heart failure with reduced ejection fraction (HFrEF), regardless of diabetes status [13,51,61].

The mechanisms underlying the cardiovascular benefits of SGLT2 inhibitors remain incompletely understood. Unlike diuretics, SGLT2 inhibitors reduce interstitial fluid without significantly affecting intravascular volume, which may partially explain their efficacy across heart failure phenotypes [62]. Additionally, SGLT2 inhibitors have been shown to reverse cardiac remodeling, particularly by improving diastolic function and reducing left ventricular fibrosis [52,63,64,65]. Cardiac fibroblasts, which regulate extracellular matrix homeostasis, play a key role in structural cardiac remodeling and heart failure progression. In vitro studies have shown that empagliflozin attenuates transforming growth factor (TGF)-β1-induced fibroblast activation, suggesting a potential mechanism for its cardioprotective effects [63].

Diabetes adversely affects vascular structure, and SGLT2 inhibitors have been shown to improve endothelial dysfunction, smooth vascular muscle homeostasis, and vascular stiffness [50,66]. These effects and their anti-inflammatory and mitochondrial function-enhancing properties contribute to their cardiovascular benefits [67].

The European Society of Cardiology (ESC) developed the Systematic Coronary Risk Evaluation 2 (SCORE2) model to estimate 10-year cardiovascular event risk. SCORE2-DM classifies patients into four risk groups and integrates diabetes status into its risk assessment [26,68]. While the cardiovascular benefits of SGLT2 inhibitors are well-documented, their impact on SCORE2 risk calculations remains underexplored. In our study, patients treated with SGLT2 inhibitors showed significant improvements in SCORE2-DM results and a marked reduction in cardiovascular event risk. Additionally, there was a notable regression in cardiovascular risk categories among patients. Our study is methodologically pioneering in this regard. Despite significant improvements observed in cardiovascular risk profiles, most patients remained classified within moderate-to-high cardiovascular risk categories. This highlights the need for additional therapeutic strategies for more comprehensive cardiovascular protection. Future studies and clinical practice might benefit from combination therapies, incorporating SGLT2 inhibitors alongside agents such as GLP-1 receptor agonists, novel non-steroidal mineralocorticoid receptor antagonists (e.g., finerenone), statins, antihypertensives, and lifestyle modification interventions, thereby providing an integrated approach to mitigate residual cardiovascular risk substantially.

It is also important to consider the potential role of patient-specific characteristics in modulating treatment efficacy. Genetic predisposition, the burden and type of comorbidities, age-related metabolic differences, and baseline endothelial function may contribute to interindividual variability in response to SGLT2 inhibitor therapy. Current research highlights the potential of precision medicine, where genetic determinants may play an essential role in predicting individual treatment responses and optimizing clinical outcomes in patients with type 2 diabetes [69]. Personalized therapeutic strategies that integrate individual-level determinants may enhance treatment precision and outcomes.

## 5. Conclusions

This study confirms that SGLT2 inhibitor therapy leads to significant reductions in cardiovascular risk scores, metabolic dysregulation, and laboratory abnormalities in patients with T2DM. The observed decreases in BMI, blood pressure, HbA1c, and triglycerides, along with stable renal function, reflect the broad therapeutic potential of these agents. The GEE analysis demonstrated robust shifts in cardiovascular risk stratification, supporting the efficacy of SGLT2 inhibitors in reducing long-term cardiovascular risk. Additionally, the comparable effects of empagliflozin and dapagliflozin highlight the class-wide benefits of these drugs. While the study’s observational design limits causal inference, the findings align with previous clinical trials emphasizing cardiometabolic protection. Future large-scale, multicenter studies with more extended follow-up periods are needed to validate these results and explore the mechanisms underlying the sustained benefits of SGLT2 inhibitors.

## 6. Limitations

This study has several limitations that should be acknowledged. First, the relatively small sample size and the six-month follow-up duration may limit the findings’ generalizability and the ability to evaluate the sustainability of the observed cardiovascular benefits. Although meaningful short-term improvements were demonstrated, the long-term durability of these effects remains uncertain. Type 2 diabetes mellitus is an established driver of cardiovascular risk, significantly increasing the incidence of cardiovascular disease compared to the general population across all age groups [70]. Therefore, more extensive multicenter trials with longer follow-up periods are warranted to validate and expand upon these findings.

Second, while the study demonstrated significant improvements in cardiovascular risk scores and metabolic parameters, observational design restricts the ability to establish causal relationships. Although patients were monitored closely, potential confounding factors, such as dietary habits, physical activity, and medication adherence, were not systematically controlled or documented, which may have influenced the outcomes.

Third, while the SCORE2-DM risk model is validated and widely recommended for cardiovascular risk estimation in patients with type 2 diabetes, it does not incorporate emerging cardiometabolic risk markers such as chronic inflammation, oxidative stress, or endothelial dysfunction—factors that are increasingly recognized as central to atherosclerotic progression. As a result, the model may underestimate the residual cardiovascular risk in specific individuals. Traditional risk prediction tools can be further enhanced by incorporating genomic information, which may provide more precise coronary artery disease (CAD) risk assessments and aid in primary prevention strategies [71].

Fourth, although missing data were handled using full-information maximum likelihood estimation under the assumption that data were missing at random (MAR), no formal sensitivity analysis was performed to test the robustness of this assumption. While FIML is a robust and commonly accepted method in clinical research, future studies may benefit from complementary approaches such as multiple imputation or Bayesian modeling to assess the potential impact of non-random missingness [72].

Lastly, the study population consisted predominantly of patients managed within a single tertiary care center, potentially limiting the applicability of findings to diverse clinical settings and populations. Differences in baseline cardiovascular risk, healthcare access, and lifestyle factors may influence the observed treatment effects.

These limitations highlight the need for further research to comprehensively assess the long-term cardiometabolic benefits of SGLT2 inhibitors across various patient populations and risk profiles.

## Figures and Tables

**Table 1:** The comparison of the 0th, 3rd, and 6th months demographic and laboratory values.

	0 Months		3rd Months		6th Months		P1	P2	P3
	Mean ± SD	Median (min–max)	Mean ± SD	Median (min–max)	Mean ± SD	Median (min–max)			
**Age, years**	58.47 ± 9.86	58(38–79)							
**Gender, n(%)**									
**Male**	80 (52.6)								
**Female**	72 (47.4)								
**Education, n (%)**									
**Literate**	12 (7.9)								
**Primary**	91 (59.9)								
**High School**	33 (21.7)								
**University**	16 (10.5)								
**Smoking, n (%)**	96 (63.2)								
**Height, cm**	165.87 ± 9.17	165 (149–184)							
**Weight, kg**	86.57 ± 11.43	85.5 (63–114)	84.88 ± 10.75	86 (63–105)	83.01 ± 9.59	83.5 (62–101)	<0.001	<0.001	<0.001
**BMI, kg/m^2^**	31.59 ± 4.45	31.25 (23.2–46.75)	30.99 ± 4.33	30.83 (22.84–45.45)	30.32 ± 4.04	29.61 (22.28–42.85)	<0.001 *	<0.001 *	<0.001 *
**SBP, mmHg**	131.64 ± 8.15	130 (110–155)	126.43 ± 6.93	125 (110–145)	122.43 ± 8.14	125 (100–145)	<0.001 *	<0.001 *	<0.001 *
**DBP, mmHg**	82.04 ± 6.49	80 (60–95)	77.53 ± 6.18	80 (60–93)	75.88 ± 6.82	75 (55–91)	<0.001	0.003	<0.001
**HbA1c, %**	9.1 ± 1.6	9.05 (6.8–12.9)	8.26 ± 1.25	8.2 (5.7–11)	7.72 ± 1.04	7.7 (5.8–11)	<0.001	<0.001	<0.001
**FBG, mg/dL**	218.78 ± 76.4	199.5 (96–441)	180.22 ± 49.03	171 (111–343)	162.39 ± 35.27	154.5 (99–302)	<0.001	<0.001	<0.001
**Urea, mg/dL**	33.81 ± 7.79	33 (16–64)	34.15 ± 7.14	34 (15–55)	34.17 ± 8.02	33 (16–56)	0.475	0.746	0.625
**Creatinine, mg/dL**	0.89 ± 0.14	0.9 (0.57–1.24)	0.96 ± 0.17	0.93 (0.6–1.4)	0.95 ± 0.22	0.98 (0.55–1.8)	<0.001	0.395	<0.001
**AST, IU/L**	35.01 ± 9.87	35 (11–59)	33.52 ± 7.88	34 (15–51)	32.21 ± 6.63	33 (16–45)	<0.001	<0.001	<0.001
**ALT, IU/L**	33.55 ± 7.02	34 (14–45)	34.34 ± 6.1	35 (15–44)	34.62 ± 5.8	35 (15–45)	0.014	0.308	0.001
**Triglycerides, mg/dL**	173.84 ± 57.6	165.5 (82–367)	159.23 ± 47.81	154 (78–340)	150.93 ± 37.13	144 (84–301)	<0.001	<0.001	<0.001
**HDL, mg/dL**	43.93 ± 8.58	42 (28–73)	44.42 ± 8.02	42 (33–70)	44.46 ± 7.49	42 (33–68)	0.052	0.707	0.298
**LDL, mg/dL**	126.07 ± 29.07	131 (49–191)	116.23 ± 23.04	116 (61–168)	111.8 ± 21.43	110 (70–159)	<0.001 *	<0.001 *	<0.001 *
**SCORE-2 DM**	21.68 ± 9.83	20.95 (2.8–49.7)			17.43 ± 8.72	16.35 (2.7–40.5)			<0.001

BMI: body mass index, SBP: systolic blood pressure, DBP: diastolic blood pressure, FBG: fasting blood glucose, AST: aspartate transaminase, ALT: alanine transaminase, HDL: high-density lipoprotein, LDL: low-density lipoprotein. P1: 3rd vs. 0th months. P2: 6th vs. 3rd months. P3: 6th vs. 0th months. *: repeated-measures analysis of variance (ANOVA), the others are compared with the Friedman test, Data presented as mean ± standard deviation (SD).

**Table 3:** Cardiovascular risk transitions within six months.

6th Month			
Low Risk	Moderate Risk	High Risk	Very High Risk
5	0	0	0
3	11	0	0
0	17	36	0
0	0	28	52

Mc-Nemar—Bowker χ^2^ = 48.00, *p* < 0.001.

**Table 4:** Post hoc analyses of the first and last measurement comparisons of DM patients.

	Adj *p*
Low Risk–Moderate Risk	0.75
Low Risk–High Risk	1
Low Risk–Very High Risk	1
Moderate Risk–High Risk	<0.001
Moderate Risk–Very High Risk	1
High Risk–Very High Risk	<0.001

**Table 5:** Very-High Risk and High-Moderate Risk comparisons.

	6th Month		*p*
0th month	High Risk	Very High Risk	<0.001
High Risk	36	0	
Very High Risk	28	52	
	Moderate Risk	High Risk	<0.001
Moderate Risk	11	0	
High Risk	17	36	

**Table 6:** General Estimating Equation (GEE) parameter estimates on Score2-DM.

Parameters	β	SE	95% Confidence Interval	Wald (χ^2^)	*p*
**Threshold**					
Score2-DM (1 = High Risk)	−0.777	0.219	(−1.206: −0.347)	12.555	<0.001
Score2-DM (2 = Low Risk)	−0.597	0.220	(−1.029: −0.165)	7.337	0.007
Score2-DM (3 = Moderate Risk)	−0.026	0.232	(−0.482: −0.429)	0.013	0.910
**Main effects**					
Measurement (Before)	−0.724	0.222	(−1.159: −0.288)	10.624	0.001
Measurement (After)					
Drug (0 = Empagliflozin)	−0.031	0.317	(−0.652: 0.590)	0.010	0.922
Drug (1 = Dapagliflozin)					
**Interaction**					
Measurement (Before) × Drug (0 = Empagliflozin)	0.374	0.309	(−0.232: 0.980)	1.462	0.227
Measurement (Before) × Drug (1 = Dapagliflozin)	0	-	-	-	-
Measurement (After) × Drug (0 = Empagliflozin)	0	-	-	-	-
Measurement (After) × Drug (1 = Dapagliflozin)	0	-	-	-	-

Test of Model effects; Measurement: *p* =< 0.001, Drug: *p* = 0.554, Measurement × Drug *p* = 0.227.

## Data Availability

The data supporting the findings of this study are not publicly available due to privacy and ethical restrictions, but may be made available by the corresponding author upon reasonable request.
