# Correction: Yalçın et al. Impact of SGLT2 Inhibitors on Cardiovascular Risk Scores, Metabolic Parameters, and Laboratory Profiles in Type 2 Diabetes. *Life* 2025, *15*, 722

**DOI:** 10.3390/life16061021

**Published:** 2026-06-18

**Authors:** Nazif Yalçın, Selman Aktaş, Seyit Uyar, Nizameddin Koca

**Affiliations:** 1 Department of Internal Medicine, Bursa Faculty of Medicine, University of Health Sciences, Bursa City Training and Research Hospital, Bursa 16009, Türkiye; nazifyalcin16@gmail.com; 2 Department of Biostatistics, Hamidiye Faculty of Medicine, University of Health Sciences, Istanbul 34396, Türkiye; selmanakts@gmail.com; 3 Department of Internal Medicine, University of Health Sciences, Antalya Training & Research Hospital, Antalya 07058, Türkiye; seyituyar79@hotmail.com

**Text Correction**

There was an error in the original publication [1]. Two sentences in Paragraph 2 of the Introduction contained inaccurate mechanistic descriptions and imprecise numerical values.

A correction has been made to Section 1 Introduction, Paragraph 2:

These drugs, also known as gliflozins or glucoretics, reduce glucose reabsorption in the renal proximal tubules, thereby lowering blood glucose levels [4]. In diabetic patients, the glucose excretion threshold is elevated due to glucose-mediated proximal tubule hypertrophy and increased SGLT2 expression [5]. These changes result in enhanced sodium (Na^+^) reabsorption, increased caloric retention, volume expansion, and subsequent hypertension [6,7]. By inhibiting SGLT2, the tubular maximum reabsorption rate is reduced, increasing urinary glucose excretion and lowering plasma glucose concentrations [8].

There was an error in the original publication [1]. The characterization of background medication effects in the Intervention paragraph was imprecise and overstated.

A correction has been made to Section 2.6 Intervention:

Patients received continuous SGLT2 inhibitor therapy (empagliflozin or dapagliflozin) as part of their antidiabetic regimen for 6 months, with clinical and laboratory assessments conducted at baseline, 3 months, and 6 months. To ensure isolated evaluation of the SGLT2 inhibitor effects, no adjustments were made to patients’ existing antihyperlipidemic, antidiabetic (excluding the newly initiated SGLT2i), or antihypertensive treatments during the study period. While drug regimens remain constant, we acknowledge that cardiovascular drugs such as antihypertensives may also have metabolic effects [27]. Patients requiring additional medical interventions during follow-up were excluded from the study.

There was an error in the original publication [1]. The concluding sentences of the GEE discussion paragraph contained an imprecise attribution of the anti-inflammatory mechanism and an incorrect single-reference citation for cardiovascular outcome data.

A correction has been made to Section 4 Discussion, Paragraph 8 (GEE analysis paragraph):

The GEE analysis revealed significant temporal reductions in cardiovascular risk categories over the six-month treatment period. Patients demonstrated a significant reduction in high-risk categorization (β = −0.777, *p* < 0.001) and low-risk categorization (β = −0.597, *p* = 0.007), with the overall effect of measurement time being highly significant (Wald χ^2^ = 10.624, *p* = 0.001). These findings suggest that SGLT2 inhibitor therapy effectively shifts patients into lower cardiovascular risk groups, which is consistent with reports that these agents reduce both systolic and diastolic blood pressure, improve glycemic control [13,50]. SGLT2 inhibitors are also associated with better outcomes in cardiovascular events [50,51].

There was an error in the original publication [1]. References [53–57] cited in the cardiovascular mechanisms paragraph were replaced with more specific and up-to-date sources directly pertaining to SGLT2 inhibitor-mediated anti-inflammatory and mitochondrial effects. The accompanying descriptive sentences were updated accordingly.

A correction has been made to Section 4 Discussion, Paragraph 10 (cardiovascular mechanisms paragraph):

Several mechanisms may underlie the observed reduction in cardiovascular risk. SGLT2 inhibitors exert protective effects beyond glycemic control by modulating endothelial function, attenuating oxidative stress, and reducing low-grade inflammation. As shown in Figure 2, SGLT2 inhibition–induced glycosuria and natriuresis lead to caloric loss and plasma-volume changes, reducing visceral adiposity, enhancing insulin sensitivity and endothelial function, ultimately translating into lower cardiovascular events and mortality. This anti-inflammatory action is supported by studies demonstrating that SGLT2 inhibitors modulate the PI3K/Akt signaling pathway, a key regulator of insulin sensitivity and inflammatory homeostasis [54]. Furthermore, recent clinical and preclinical evidence indicates that SGLT2 inhibitors exert potent immunomodulatory effects by suppressing pro-inflammatory cytokines and chemokines, thereby significantly improving systemic metabolic outcomes [55]. These pathways may partially explain our cohort’s observed reductions in cardiovascular risk scores. These cardioprotective effects involve mitochondrial stabilization and suppression of pro-inflammatory pathways. SGLT2 inhibitors have been shown to enhance mitochondrial oxidative phosphorylation and ATP production while simultaneously reducing reactive oxygen species (ROS) generation, thereby playing a pivotal role in maintaining mitochondrial integrity and improving overall metabolic function [56,57].

There was an error in the original publication. The description of selenoproteins in the emerging therapies paragraph overstated their established properties and made an unsupported claim regarding complementarity with SGLT2 inhibitors.

A correction has been made to Section 4 Discussion, Paragraph 11 (future directions/emerging therapies paragraph):

Beyond currently available pharmacological options, future research may explore the synergistic potential of combining SGLT2 inhibitors with emerging therapeutic agents. For example, selenoproteins are established modulators of insulin sensitivity, and while their potential synergistic effects when combined with SGLT2 inhibitor therapy remain to be fully investigated, they represent a promising area for multi-dimensional metabolic research [58]. Additionally, extracellular vesicles derived from stem cells have shown promise in enhancing endothelial repair, modulating immune responses, and promoting tissue regeneration—pathways that align with the cardioprotective profile of SGLT2 inhibitors [59]. Integrating novel adjuvant therapies with SGLT2 inhibition could represent a forward-looking, multidimensional approach to metabolic and cardiovascular risk reduction.

There was an error in the original publication. Reference [69] cited in the patient-specific characteristics paragraph was replaced with a more directly relevant precision medicine source. The accompanying sentence was updated to align with the new citation.

A correction has been made to Section 4 Discussion, Paragraph 16 (patient-specific characteristics paragraph):

It is also important to consider the potential role of patient-specific characteristics in modulating treatment efficacy. Genetic predisposition, the burden and type of comorbidities, age-related metabolic differences, and baseline endothelial function may contribute to interindividual variability in response to SGLT2 inhibitor therapy. Current research highlights the potential of precision medicine, where genetic determinants may play an essential role in predicting individual treatment responses and optimizing clinical outcomes in patients with type 2 diabetes [69]. Personalized therapeutic strategies that integrate individual-level determinants may enhance treatment precision and outcomes.

There was an error in the original publication. Reference [70] in the first limitations paragraph cited an epidemiological projection study that was not directly relevant to the statement being made. It was replaced with a more appropriate reference supporting the cardiovascular risk associated with T2DM. The accompanying sentence was updated accordingly.

A correction has been made to Section 6 Limitations, Paragraph 1:

This study has several limitations that should be acknowledged. First, the relatively small sample size and the six-month follow-up duration may limit the findings’ generalizability and the ability to evaluate the sustainability of the observed cardiovascular benefits. Although meaningful short-term improvements were demonstrated, the long-term durability of these effects remains uncertain. Type 2 diabetes mellitus is an established driver of cardiovascular risk, significantly increasing the incidence of cardiovascular disease compared to the general population across all age groups [70]. Therefore, more extensive multicenter trials with longer follow-up periods are warranted to validate and expand upon these findings.

There was an error in the original publication. A minor grammatical redundancy was present in the second paragraph of the Limitations (“the applied observational design” was replaced with “observational design”).

A correction has been made to Section 6 Limitations, Paragraph 2:

Second, while the study demonstrated significant improvements in cardiovascular risk scores and metabolic parameters, observational design restricts the ability to establish causal relationships. Although patients were monitored closely, potential confounding factors, such as dietary habits, physical activity, and medication adherence, were not systematically controlled or documented, which may have influenced the outcomes.

There was an error in the original publication. Reference [71] in the third limitations paragraph cited an unrelated machine-learning analysis. It was replaced with a landmark genomic risk prediction study directly supporting the claim about genomic information in CAD risk assessment. The accompanying sentence was updated accordingly.

A correction has been made to Section 6 Limitations, Paragraph 3:

Third, while the SCORE2-DM risk model is validated and widely recommended for cardiovascular risk estimation in patients with type 2 diabetes, it does not incorporate emerging cardiometabolic risk markers such as chronic inflammation, oxidative stress, or endothelial dysfunction—factors that are increasingly recognized as central to atherosclerotic progression. As a result, the model may underestimate the residual cardiovascular risk in specific individuals. Traditional risk prediction tools can be further enhanced by incorporating genomic information, which may provide more precise coronary artery disease (CAD) risk assessments and aid in primary prevention strategies [71].

 

**References**

With this correction, references [4,9–11,18,27,53–57,60,66,68–72] have been replaced with more appropriate and up-to-date sources.

4. Kramer, C.K.; Zinman, B. Sodium-Glucose Cotransporter-2 (SGLT-2) Inhibitors and the Treatment of Type 2 Diabetes. *Annu. Rev. Med.* **2019**, *70*, 323–334. https://doi.org/10.1146/annurev-med-042017-094221.
9. Downs, C.A.; Faulkner, M.S. Toxic stress, inflammation and symptomatology of chronic complications in diabetes. *World J. Diabetes* 2015, *6*, 554–565. https://doi.org/10.4239/wjd.v6.i4.554.
10. Luc, K.; Schramm-Luc, A.; Guzik, T.J.; Mikolajczyk, T.P. Oxidative stress and inflammatory markers in prediabetes and diabetes. *J. Physiol. Pharmacol.* **2019**, *70*, 809–824. https://doi.org/10.26402/jpp.2019.6.01.
11. Yang, L.; Zhang, X.; Wang, Q. Effects and mechanisms of SGLT2 inhibitors on the NLRP3 inflammasome, with a focus on atherosclerosis. *Front. Endocrinol.* **2022**, *13*, 992937. https://doi.org/10.3389/fendo.2022.992937.
18. Talha, K.M.; Anker, S.D.; Butler, J. SGLT-2 Inhibitors in Heart Failure: A Review of Current Evidence. *Int. J. Heart Fail.* 2023, *5*, 82–90. https://doi.org/10.36628/ijhf.2022.0030.
27. Cooper-DeHoff, R.; Karnes, J. Antihypertensive medications: Benefits of blood pressure lowering and hazards of metabolic effects. *Expert Rev. Cardiovasc. Ther.* **2009**, *7*, 689–702.
53. Kim, S.R.; Lee, S.G.; Kim, S.H.; Kim, J.H.; Choi, E.; Cho, W.; Rim, J.H.; Hwang, I.; Lee, C.J.; Lee, M.; et al. SGLT2 inhibition modulates NLRP3 inflammasome activity via ketones and insulin in diabetes with cardiovascular disease. *Nat. Commun.* **2020**, *11*, 2127. https://doi.org/10.1038/s41467-020-15983-6.
54. Rykova, E.Y.; Klimontov, V.V.; Shmakova, E.; Korbut, A.I.; Merkulova, T.I.; Kzhyshkowska, J. Anti-Inflammatory Effects of SGLT2 Inhibitors: Focus on Macrophages. *Int. J. Mol. Sci.* **2025**, *26*, 1670.
55. Ullah, A.; Shen, B. Immunomodulatory effects of anti-diabetic therapies: Cytokine and chemokine modulation by metformin, sodium-glucose cotransporter 2 inhibitors, and glucagon-like peptide-1 receptor agonists (2013–2025). *Eur. J. Med. Chem.* **2025**, *299*, 118065.
56. Chambers, J.M.; Croteau, D.; Pimentel, D.R.; Gower, A.C.; Panagia, M.; Baka, T.; Qin, F.; Luptak, I.; Colucci, W.S. SGLT2 inhibitor upregulates myocardial genes for oxidative phosphorylation and fatty acid metabolism in Gαq-mice. *J. Mol. Cell. Cardiol. Plus* **2025**, *12*, 100296. https://doi.org/10.1016/j.jmccpl.2025.100296.
57. Koizumi, T.; Watanabe, M.; Yokota, T.; Tsuda, M.; Handa, H.; Koya, J.; Nishino, K.; Tatsuta, D.; Natsui, H.; Kadosaka, T.; et al. Empagliflozin suppresses mitochondrial reactive oxygen species generation and mitigates the inducibility of atrial fibrillation in diabetic rats. *Front. Cardiovasc. Med.* **2023**, *10*, 1005408. https://doi.org/10.3389/fcvm.2023.1005408.
60. Ma, C.X.; Ma, X.N.; Guan, C.H.; Li, Y.D.; Mauricio, D.; Fu, S.B. Cardiovascular disease in type 2 diabetes mellitus: Progress toward personalized management. *Cardiovasc. Diabetol.* **2022**, *21*, 74. https://doi.org/10.1186/s12933-022-01516-6.
66. Salvatore, T.; Galiero, R.; Caturano, A.; Rinaldi, L.; Di Martino, A.; Albanese, G.; Di Salvo, J.; Epifani, R.; Marfella, R.; Docimo, G.; et al. An Overview of the Cardiorenal Protective Mechanisms of SGLT2 Inhibitors. *Int. J. Mol. Sci.* **2022**, *23*, 3651. https://doi.org/10.3390/ijms23073651.
68. Mollace, R.; Nardin, M.; Arzenton, M.; Testerini, F.; Fumagalli, A.; Nicoli, F.; Licastro, M.C.; Nudi, A.; Bernardini, V.; Frascaro, F.; et al. SCORE2-diabetes for predicting coronary artery disease: A cardiac CT study in a diabetic moderate-risk region population. *Cardiovasc. Diabetol.* **2025**, *24*, 464. https://doi.org/10.1186/s12933-025-03000-3.
69. Galiero, R.; Caturano, A.; Vetrano, E.; Galiero, R.; Caturano, A.; Vetrano, E.; Monda, M.; Marfella, R.; Sardu, C.; Salvatore, T.; et al. Precision Medicine in Type 2 Diabetes Mellitus: Utility and Limitations. *Diabetes Metab. Syndr. Obes.* **2023**, *16*, 3669–3689. https://doi.org/10.2147/DMSO.S390752.
70. Booth, G.L.; Kapral, M.K.; Fung, K.; Tu, J.V. Relation between age and cardiovascular disease in men and women with diabetes compared with non-diabetic people: A population-based retrospective cohort study. *Lancet* **2006**, *368*, 29–36. https://doi.org/10.1016/S0140-6736(06)68967-8.
71. Inouye, M.; Abraham, G.; Nelson, C.P.; Wood, A.M.; Sweeting, M.J.; Dudbridge, F.; Lai, F.Y.; Kaptoge, S.; Brozynska, M.; Wang, T.; et al. Genomic Risk Prediction of Coronary Artery Disease in 480,000 Adults: Implications for Primary Prevention. *J. Am. Coll. Cardiol.* **2018**, *72*, 1883–1893. https://doi.org/10.1016/j.jacc.2018.07.079.
72. Little, R.J.; Rubin, D.B. *Statistical Analysis with Missing Data*, 3rd ed.; Wiley: Hoboken, NJ, USA, 2019.

The authors state that the scientific conclusions are unaffected. This correction was approved by the Academic Editor. The original publication has also been updated.
